# Acute Liver Injury With Cholestatic Pattern: An Adverse Effect of Trimethoprim-Sulfamethoxazole

**DOI:** 10.7759/cureus.92749

**Published:** 2025-09-19

**Authors:** Makhlouf Bannoud, Lucia Hong, Maya Ibelaidene, Milan Acosta, Chris Mehdizadeh

**Affiliations:** 1 Internal Medicine, University of California, Riverside School of Medicine, Riverside, USA; 2 Internal Medicine, University of California, Davis School of Medicine, Davis, USA; 3 Anesthesiology, University of California, Irvine Medical Center, Orange, USA

**Keywords:** bactrim, cholestasis, drug-induced liver injury, jaundice, trimethoprim-sulfamethoxazole

## Abstract

Trimethoprim-sulfamethoxazole (TMP-SMX), commonly known by the brand name Bactrim, is a combination antibiotic medication that is used to treat a broad spectrum of infections. It is known to be associated with a wide array of adverse reactions, including drug-induced liver injury (DILI). Drug-induced liver injury associated with TMP-SMX more commonly presents with a hepatocellular or mixed liver injury pattern rather than a cholestatic pattern. Here, we present the case of a 52-year-old female patient who presented with jaundice and pruritus after a recent course of TMP-SMX. Labs and imaging revealed cholestatic liver injury without obstruction. Further testing ruled out autoimmune and infectious causes. The patient’s condition was resolved within five days of presentation with only supportive care administered. Given the clinical course as well as the exclusion of other likely causes, we concluded that this was a case of TMP-SMX-associated acute liver injury with a cholestatic pattern.

## Introduction

Trimethoprim-sulfamethoxazole (TMP-SMX) is a combination antibiotic with broad-spectrum bacteriostatic activity that works by inhibiting folate synthesis. Specifically, trimethoprim is a direct competitor of dihydrofolate reductase, while sulfamethoxazole is a direct competitor of p-aminobenzoic acid. Common adverse effects of TMP-SMX include nausea, vomiting, skin rash, and pruritus. More serious adverse effects include renal tubular acidosis, hepatitis, hypoglycemia, hyponatremia, megaloblastic anemia, and hemolysis in glucose-6-phosphate dehydrogenase (G6PD) deficiency. Older and immunocompromised patients may be at risk of life-threatening consequences such as neutropenia, anaphylaxis, and Stevens-Johnson Syndrome [1]. Although liver injury is a relatively well-known and well-documented potential sequelae of TMP-SMX use, its presentation may vary from hepatocellular injury to mixed liver injury to cholestatic injury, and severity may vary from transient acute hepatitis to fulminant liver failure requiring transplant [2,3]. The acute onset of jaundice with no other plausible cause in the setting of recent TMP-SMX use should raise a strong suspicion for drug-induced liver injury, and the appropriate workup should be conducted to reduce mortality in these patients.

This report was previously presented as a meeting abstract at the 2023 American College of Physicians California Southern Regions 1, 2 & 3 Annual Scientific Meeting on October 7, 2023.

## Case presentation

A 52-year-old female patient with no significant past medical history presented for evaluation of scleral icterus for three days. She had been recently diagnosed with a urinary tract infection in Mexico and was prescribed an 11-day course of metronidazole, TMP-SMX, and phenazopyridine that she had completed the day before presentation. Of note, the exact doses of metronidazole, TMP-SMX, and phenazopyridine prescribed in Mexico were not available in the patient’s records and could not be confirmed. She reported no other symptoms during the medication course except itching of the hands and feet.

**Figure 1:**
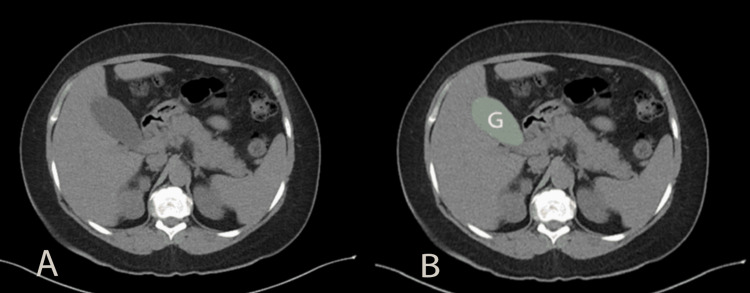
CT abdomen showing gallbladder distention A: Unlabeled axial view demonstrating the gallbladder in situ; B: Axial view with the gallbladder highlighted in green (labeled 'G') for clarity

**Figure 2:**
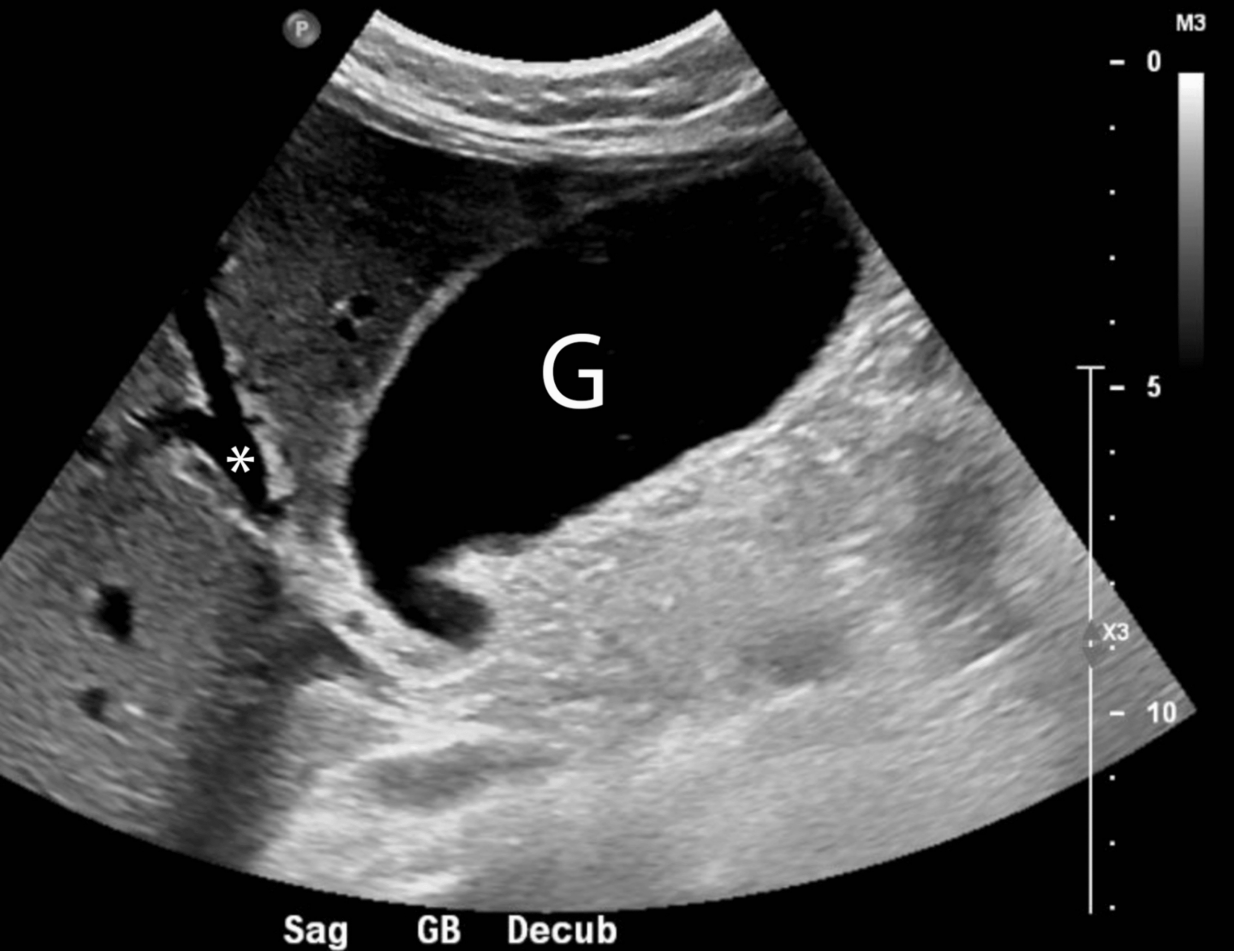
Right upper quadrant ultrasound The ultrasound demonstrates distention of the gallbladder (G) with no obvious obstruction. The common bile duct is measured to be 5.4mm in diameter (*).

On presentation, her vitals were within normal limits. Her initial labs were notable for the following (Table 1): WBC 6,400 cells/µL, hemoglobin (Hgb) 12.1 g/dL, hematocrit (Hct) 35.5%, platelets 451,000 cells/µL, total bilirubin 7.8 mg/dL (direct 6.3 mg/dL), alanine aminotransferase (ALT) 123 U/L, aspartate aminotransferase (AST) 102 U/L, alkaline phosphatase (ALP) 903 U/L, lipase 215 U/L. A CT abdomen (Figure 1) showed nonspecific gallbladder distention with no hepatomegaly, masses, abscesses, or other causes of acute liver injury. Right upper quadrant ultrasound (Figure 2) demonstrated a distended gallbladder with borderline wall thickening, minimal sludge, and a negative sonographic Murphy sign with no biliary obstruction and a common bile duct diameter of 5.4 mm. Together, these findings indicated cholestatic hepatitis with no obstruction in the biliary tree.

**Table 1:** Laboratory evaluation at the time of presentation (day one), day two, and day five Hgb: Hemoglobin, Hct: Hematocrit, ALT: Alanine aminotransferase, AST: Aspartate aminotransferase, ALP: Alkaline phosphatase

Lab test	Day one	Day two	Day five	Normal reference range
WBC	6,400 cells/µL	—	—	4,000-11,000 cells/µL
Hgb	12.1 g/dL	—	—	13.5-17.5 g/dL (male), 12-15.5 g/dL (female)
Hct	35.5%	—	—	38-50% (male), 34.9-44.5% (female)
Platelets	451,000 cells/µL	—	—	150,000-450,000 cells/µL
Total bilirubin	7.8 mg/dL	6.4 mg/dL	3.3 mg/dL	0.1-1.2 mg/dL
Direct bilirubin	6.3 mg/dL	—	—	0-0.3 mg/dL
ALT	123 U/L	144 U/L	282 U/L	7-56 U/L
AST	102 U/L	153 U/L	157 U/L	10-40 U/L
ALP	903 U/L	939 U/L	—	44-147 U/L
Lipase	215 U/L	—	—	0-160 U/L

On day two of admission, liver enzymes continued to trend upward, but bilirubin began trending down: ALT 144 U/L, AST 153 U/L, ALP 939 U/L, total bilirubin 6.4 mg/dL (Table 1). To investigate possible causes of this cholestatic liver injury, autoimmune and infectious etiologies were considered and ruled out. The IgG-4, anti-mitochondrial antibodies, antinuclear antibodies, and viral hepatitis panel were all within normal limits.

On day three of admission, liver enzymes started trending down, and bilirubin continued to decrease as well. The patient was discharged on day five with clinical resolution of her initial complaint of jaundice and labs as follows: bilirubin 3.3 mg/dL, ALT 282 U/L, AST 157 U/L (Table 1). The patient was managed with supportive therapy only, consisting of monitoring and symptomatic care without steroids or other specific interventions. Given that her symptoms resolved with supportive treatment alone, drug-induced cholestasis was presumed to be the most likely diagnosis. Thus, TMP-SMX was the most likely culprit, as it is known to cause liver toxicity.

## Discussion

Cholestatic liver injury is characterized by impaired bile flow, leading to the accumulation of bile acids within hepatic tissue and systemic circulation. It typically presents with elevated serum levels of alkaline phosphatase and bilirubin. Clinically, patients may experience jaundice, pruritus, and acholic stools. TMP-SMX is a widely used antibiotic that has been implicated in various forms of drug-induced liver injury (DILI), including the cholestatic pattern observed in this case. While the patient had also received metronidazole and phenazopyridine, these agents are less commonly associated with clinically significant cholestatic liver injury and were therefore considered less likely contributors to the presentation.

The TMP-SMX hypersensitivity reactions often begin with a prodrome of fever and rash prior to the onset of jaundice. However, atypical cases, such as the one described here, may lack this prodromal phase. Trimethoprim alone has been associated with mixed or cholestatic liver injury without overt immunoallergic features, which may represent the underlying mechanism in our patient. Although the exact pathogenesis remains unclear, proposed mechanisms include immune-mediated hypersensitivity and the formation of toxic or antigenic metabolites [4].

Drug-induced liver injury can result from a wide array of substances, including prescription medications, over-the-counter drugs, and herbal supplements, through diverse pathways. Trimethoprim-sulfamethoxazole has previously been linked to both mild and severe hepatic outcomes, ranging from transient cholestasis to vanishing bile duct syndrome and fulminant hepatic failure. More commonly, the injury appears to stem from metabolite-related toxicity, particularly through glutathione-mediated pathways [5-7]. The risk of hepatotoxicity is further amplified by variations in cytochrome P450 isoenzyme activity and acetylation rates, which can influence the generation and clearance of toxic intermediates [8]. In many reported cases, hypersensitivity features, such as fever, rash, and eosinophilia, prompted early recognition and discontinuation of the drug [8-10]. However, this case highlights the importance of recognizing that TMP-SMX-induced liver injury may occur even in the absence of such constitutional signs.

Several prior reports have documented protracted cholestatic liver injury secondary to TMP-SMX. Faria et al. described a case of severe, prolonged cholestasis following a five-day course of TMP-SMX. Liver biopsy confirmed the presence of bile within hepatocytes and bile casts in hepatic ducts. The patient had a clinical and biochemical profile similar to our case but required 35 months for resolution, managed symptomatically with cholestyramine and hydroxyzine [11].

In another report, Muñoz et al. described a case of intrahepatic cholestasis lasting eight months, associated with phospholipidosis on serial liver biopsies. This finding was likely attributable to lipid-soluble metabolites and inhibition of phospholipase A1. The patient’s symptoms improved only after exchange plasmapheresis, suggesting a role for circulating toxic-lipid complexes [12]. These reports emphasize the potential for TMP-SMX to cause severe, prolonged cholestatic reactions in certain individuals and the importance of early recognition.

Our case adds to the literature by illustrating a self-limited cholestatic liver injury induced by TMP-SMX in the absence of classic hypersensitivity features. It underscores the importance of a detailed medication history and timely diagnostic workup in patients presenting with new-onset jaundice, even when constitutional symptoms are absent.

## Conclusions

This case highlights an atypical presentation of TMP-SMX-induced cholestatic liver injury in a previously healthy woman, occurring without the classic prodrome of fever, rash, or eosinophilia. Recognition was made possible through a detailed medication history and exclusion of other causes. Prompt discontinuation of the offending drug and supportive management led to full recovery. Clinicians should maintain a high index of suspicion for drug-induced liver injury in patients with new-onset jaundice or cholestasis, even in the absence of systemic symptoms, particularly when recent antibiotic exposure is identified.
